# Electrical and antimicrobial studies of multifunctional ZrO_2_ doped borate glasses and glass ceramics

**DOI:** 10.1038/s41598-025-27740-0

**Published:** 2025-11-26

**Authors:** Gehad Y. Abo El-Reesh, M. A. Azooz, M. A. Ouis, Amira A. Gamal, S. M. Abbas, Ragab M. Mahani, Azza Ward, S. Abd Elkhalik

**Affiliations:** 1 Chemistry Department, Faculty of Science, Beni-Suef University, Beni-Suef, Egypt; 2 Glass Research Department, National Research Centre, Dokki, Giza, Egypt; 3 Chemistry of Natural and Microbial Products Department, Pharmaceutical and Drug Industries Research Institute, National Research Centre, Dokki, Cairo, Egypt; 4 Microwave Physics and Dielectrics Department, National Research Centre, Dokki, Cairo, 12622 Egypt

**Keywords:** Borate glass, ZrO_2_, Glass–ceramic, Permittivity, Loss tangent, Antimicrobial activity, Chemistry, Materials science, Nanoscience and technology

## Abstract

In this study, borate-based glass and glass–ceramics doped with varying concentrations of ZrO_2_ were synthesized, followed by controlled heat treatment for crystallization. X-ray diffraction, density, Fourier-transform infrared spectroscopy, field emission scanning electron microscope, and dielectric relaxation spectroscopy were employed to characterize the prepared samples. XRD analysis confirmed the formation of the nanocrystalline ZrO_2_ phase within the glass–ceramic matrix. The average crystallite size, determined by the Scherrer formula, fell within the nanometric range. DRS investigated the dielectric response of all samples over a wide frequency range (0.1 Hz–1 MHz) at 30 °C. It showed enhanced dielectric properties with increasing ZrO_2 _content. Significantly increased permittivity while decreased loss tangent for glass and glass ceramics with higher ZrO_2 _content. Furthermore, glass ceramic exhibited better dielectric properties than glass samples. For electrical properties, the optimal mol% suggested for Zr^4+^ is 2 since it exhibited the highest permittivity (~ 23 at 1 MHz) and lowest loss tangent (~ 0.005) for glass and glass ceramics. The substitution of CaO by ZrO_2 _increases both permittivity and AC conductivity while reducing the dielectric losses, confirming the enhancement of dielectric properties. Furthermore, the antimicrobial activity of the prepared samples was tested. The antimicrobial activity of the glass–ceramic results from the presence of ZrO_2 _nanocrystals, which act in a similar manner to ZrO_2_ nanoparticles. Cytotoxicity and long-term stability assays were carried out. The results display the effect of ZrO_2_ on structure, crystallinity, and the noticed electrical and biological responses, making them promising materials for use in applications that require electrical functionality and biocompatibility.

## Introduction

Borate glasses are considered wide glass formers that have been employed in many applications, including high-power solid-state lasers, smart window manufacturing, ionic conductors, radioactive waste immobilization, agri-glass for plant nutrition, and bioactive materials^1–6^. Being bioactive materials, Glass can be used as dental cements, toothpaste, composites, synthetic scaffolds, treating burns and wounds, and bioactive antibacterial materials^7–9^. Increasing the rate of antibiotic-resistant microorganisms calls for finding new ways and materials with antimicrobial properties to minimize the excessive use of antibiotics^10–14^. Bioactive glasses can be considered as one of the most important types of biomaterials that can represent combinations of properties, including bioactivity, biocompatibility, and antimicrobial activity through the release of antimicrobial agents that can overcome infections^15,16^. Silicate glasses show great effect in biomedical applications; however, their lower solubility rate reduces their reaction and compatibility with biological tissues^17^. Unlike silicate glasses, lower chemical durability and fast dissolution rates of borate glasses make them more reactive than silicate glasses and suitable for biological applications^18^. Due to their antimicrobial properties, some ions such as Ag^+^, Cu^2+^, Zn^2+^, and Zr^4+^ are often incorporated into biomaterials to overcome and reduce infections by inhibiting the microbial growth upon the release of these ions from biomaterials^19–21^. Also, borate glasses have the flexibility for doping with several antimicrobial metal ions (such as Ag^+^, Cu^2+^, Zn^2+^, and Zr^4+^) for reducing the risk of infection spread^22,23^. Recently, antimicrobial ceramics have gained significant interest due to their mechanical properties, long-term antibacterial activity, good bioactivity, and biocompatibility^12,24,25^. To improve the glass and glass ceramics structure, electrical, and biological properties, several studies have been employed, especially through the use of different additives^26^. da Silva et al. studied the effect of Ag_2_O-doped borate glass on in vitro bioactivity, cytotoxicity, and antibacterial properties against E. coli and Staphylococcus aureus; the results indicate the great potential of the materials to be used as repairs for living tissues^27^. Also, Demirel and Taygun studied the physical, mechanical, thermal, and antibacterial properties of 5% ZnO-doped borosilicate glass and glass–ceramic system; the results indicate a remarkable product for the pharmaceutical industry (drug packaging)^28^. Among these, ZrO_2_-added glass and glass ceramics have represented promising findings^29–34^. In medical fields, zirconia acquired significant attention depending on its bioactivity, biocompatibility, and mechanical properties^35^, and it is widely used as a dental crown^24^, bone implants^36^, and femoral heads for total hip replacement ^37^. Additionally, ZrO_2_ has been shown to have improved antimicrobial properties against various microbial species^38,39^. By using a combination of XRD and TEM techniques, Kleebusch et al. succeeded in describing the crystallization of ZrO_2_ nanocrystals at 725 °C with time proceeding in Li_2_O /Al_2_O_3_/SiO_2_ system through the formation of Zr-rich liquid–liquid phase separation droplets, which finally evolved into crystalline ZrO_2_^40^. Recently, nanoparticles have attracted great attention in biomedical fields related to their novel properties, including unique size, distribution, and morphology. ZrO_2_ nanoparticles can be employed as antioxidant, anticancer, and antimicrobial materials^41,42^. The antimicrobial results obtained by Bahammam et al. demonstrated that nano-ZrO_2_ effectively reduces the inhibition zone for both fungal and bacterial activities^43^. In borate glasses, BO_3_ and BO_4_ units create a network made of a B_2_O_3_ network. Upon introducing calcium oxide (CaO) or lithium fluoride (LiF), some of the connections between boron and oxygen can break, creating non-bridging oxygens (NBOs), thus affecting electrical and structural properties^44^. In contrast, Al_2_O_3_ often enters as a network former, increasing cross-linking between boron and oxygen, i.e., reducing NBOs and rigidity, thus raising glass transition temperature (T_g_) and reducing ionic conductivity. Upon doping with ZrO_2_, high-field-strength Zr^4+^ ions are introduced that further crosslink the network. When Ca–B_2_O_3_–SiO_2_ systems are doped with ZrO_2_, NBOs are reduced and network connectivity is enhanced, improving structure and dielectric stability^45^. Upon increasing CaO content in borate glasses, resistivity enhances and dielectric loss reduces due to strengthening bond networks and limiting charge migration^45^. ZrO_2_ dopants significantly enhance the dielectric properties of CaO–B_2_O_3_–SiO_2_ glass–ceramics, exploring lower losses and higher permittivity resulting from densification and reduced defects^46^. More importantly, ZrO₂-modified glass–ceramics exhibit enhanced permittivity (~ 10–20) and low tangent losses, qualifying them for low-temperature co-fired ceramic (LTCC) applications^47^. Upon treating the doped glass at specific temperatures, the glass–ceramic formed. During ceramization, ZrO₂ modifies crystallization pathways, improving hardness, lowering thermal expansion, and enhancing dielectric properties through optimized dispersion of fine crystalline phases^44^.

The aims of this present work are:

- Studying the impact of the gradual increase of ZrO_2_ mol% on structural, electrical, and biological properties of the glass system (40 B_2_O_3_–20 Al_2_O_3_–10 LiF-(30-X) CaO–X ZrO_2_).
- Characterization of the synthesized samples by various tools, including X-ray diffraction (XRD), Fourier transform infrared spectroscopy (FT-IR), Density and Molar volume, and Field emission scanning electron microscopy (FESEM).
- Investigating electrical and biological properties to provide a complete evaluation of these multifunctional materials; such a dual evaluation shows the relevance of ZrO_2_-doped borate glass and glass–ceramics for biomedical and bioelectronics applications with long-term structural stability.

## Experimental section

### Materials

The starting raw materials were used without previous purification. Orthoboric acid (H_3_BO_3_, Winlab, Leicestershire, UK, Assay 99.5%) for B_2_O_3_, Aluminium oxide (Fluka, chemei AG, switzerland), Calcium carbonate (CaCO_3_, ADWIC, Egypt) for CaO, Zirconium dioxide (Fluka, Germany) for ZrO_2_, and Lithium fluoride (Fluka, Biochemika, Assay ≥ 99%).

### Preparation of materials

#### Glass preparation

ZrO_2_-doped borate glass of the system (40 B_2_O_3_–20 Al_2_O_3_–10 LiF-(30-X) CaO–X ZrO_2_), where (0 ≤ X ≤ 2.5) was prepared via the traditional melt quenching method. The glass batches of the starting materials were accurately weighed and melted in an electric laboratory furnace at 1350 ± 5 °C for 2 h. The molten mixtures were continuously checked and rotated every 30 min for mixing and obtaining a high level of homogeneity. The molten glasses were carefully cast into pre-heated Stainless steel molds and transferred rapidly to a muffle furnace for annealing at 400 °C for 1 h, then left to cool to room temperature. The compositions in mol% of the synthesized glasses are listed in Table 1

**Table 1:** Composition of the base and ZrO_2_-doped glass in mol%.

Glass code	B_2_O_3_	Al_2_O_3_	LiF	CaO	ZrO_2_
BGZr0	40	20	10	30	–
BGZr1	40	20	10	29.5	0.5
BGZr2	40	20	10	29	1
BGZr3	40	20	10	28.5	1.5
BGZr4	40	20	10	28	2

#### Glass–ceramic preparation

The glass–ceramics were obtained from the parent glass by heat treatment. First, the glass samples were heated to nucleating temperature (500 °C) with a rate of 5 °C/min for 4 h. The temperature was then increased to the crystal growth temperature (725 °C) with a constant rate of 5 °C/min, and the samples were maintained at this temperature for 10 h. The samples were allowed to cool down to room temperature at a rate 25 °C/min. The codes assigned to the base glass–ceramic is BGCZr0, where the code of the ZrO_2_-doped glass–ceramics are BGCZr1, BGCZr2, BGCZr3, and BGCZr4, respectively.

### X-ray diffraction analysis

X-ray diffraction (XRD) profiles were recorded for glass–ceramics with a PANalytical (Empyrean) diffractometer using Cu Kα radiation (wavelength 0.154 nm) at a current of 35 mA, an accelerating voltage of 40 kV, scan angle range from 3° to 70°, and scan step of 0:02°.

### FTIR spectral analysis

The Fourier transform infrared (FT-IR) spectra (4000–400 cm^-1^) were collected by a Bruker (Vertex 70) spectrometer for glasses and glass–ceramics through the KBr technique. The pulverized KBr was mixed with powder samples with a ratio of 100:1 mg, respectively. The mixtures were compacted under a pressure of 5 tons/cm^2^ to form homogenized discs. The obtained discs were then immediately analyzed.

### Density measurements

Density was measured at room temperature by applying the standard Archimedes method; distilled water was used as an immersion liquid. The density measurements are precise to ± 0.02 g/cm^3^. Density can be calculated by using Eq. (1)

1 $$\rho = {\text{W}}_{{\text{a}}} \rho_{{\text{w}}} /\left( {{\text{W}}_{{\text{a}}} - {\text{W}}_{{\text{w}}} } \right)$$

where ρ is the density of the glass sample, ρ_w_ is the density of distilled water, W_w_ and W_a_ are the weights of the glass sample in distilled water, respectively.

The molar volume, which can be defined as the volume of one gram mole of glass, can be determined by Eq. (2)

2 $${\text{V}}_{{\text{m}}} = 1/\rho \, \sum {{\text{X}}_{{\text{i}}} {\text{M}}_{{\text{i}}} }$$

where X_i_ and M_i_ are the components of molar fraction and molecular weight.

### Field emission scanning electron microscope (FESEM) graphs

The morphology of the glass–ceramic samples was examined by SEM (Quanta FEG250, Thermo Fisher Scientific (FEI)). Before SEM examination, the samples were carefully cleaned and completely dried to avoid any outgassing in the vacuum chamber. A small amount of powder sample was mounted on an aluminum conductive stub using double-sided conductive carbon tape to ensure good electrical contact. For non-conductive samples, a thin layer of gold was deposited using a sputter coater (S150A Sputter Coater, Edwards, England) in order to prevent surface charging and improve image quality. Finally, the surface morphology of the samples was examined using a Field-Emission Scanning Electron Microscope (FESEM).

### Dielectric measurements

For dielectric measurements all glass and glass ceramic samples were prepared as polished discs with a diameter of approximately 15 mm and a thickness of ~ 4 mm. Then, their flat surfaces were coated with silver paste to ensure good electrical contact. This configuration corresponds to parallel-plate geometry, suitable for impedance spectroscopy and modulus analysis.

Dielectric and conductivity measurements were conducted using a high-resolution broadband impedance analyzer (Schlumberger Solartron 1260), together with an electrometer, amplifier, and measuring cell, as previously detailed^48^. The frequency spectrum of the applied alternating current electric field spanned from 0.1 to 1 MHz. Effective electromagnetic shielding was applied to the whole sample holder to mitigate noise issues, particularly at low frequencies. The measurements were automated by connecting the impedance analyzer to a personal computer using a GPIB cable (IEEE 488). The commercial interface and automation program LabVIEW was utilized for data acquisition. The errors in ε' and ε'' are 1% and 3%, respectively. The samples’ temperature was regulated by a temperature controller utilizing a Pt 100 sensor. The inaccuracy in temperature measurements is 0.5 °C. The samples were stored in desiccators with silica gel to prevent moisture. Subsequently, the sample was placed in the measuring cell and allowed to remain with P_2_O_5_ until the measurements were conducted.

### Biological effects experiments

#### Microbial strains

To study the antimicrobial activity of the samples, five types of microorganisms were subjected to testing. The clinical strains of one G-ve bacteria strain *(Escherichia coli)*, two G + ve strains (*Staphylococcus aureus*, *Bacillus cereus*), one fungus (*Aspergillus niger)*, and one yeast (*Candida albicans*) collected from a biobank of El-Demerdash hospital (Cairo, Egypt) under accession numbers:

*Staphylococcus aureus* (25,923), *Bacillus cereus* (33,018), *Escherichia coli* (8739), *Aspergillus niger* (EM77), and *Candida albicans* (10,231).

#### Inoculum preparation

The used media were subjected to sterilization for inoculum preparation; the nutrient broth medium and the potato dextrose broth medium were used for bacteria and fungi and yeasts, respectively. The bacteria-containing tubes were incubated for 24 h at 37 °C, while those of yeast and fungi were incubated for 2–3 days at 30 °C.

#### Determination of antimicrobial assay

The ten glass and glass–ceramic samples were in vitro tested for their antimicrobial activity. Clinically standard isolated microorganism strains were employed for the determination of antimicrobial activity, such as *Staphylococcus aureus* and *Bacillus cereus* (Gram-positive bacteria), *E. coli* (Gram-negative bacteria), *Aspergillus niger* (fungi), and *Candida albicans* (yeast), by measuring the growth inhibition of pathogenic organisms according to^49^. 0.03 g of samples were loaded into the tubes [10 mL of nutrient broth (bacteria and yeast) or potato dextrose broth (fungi)]. The inoculated tubes (100 µl from the inoculum) and control tubes (without samples) were incubated for 24 h at the optimum temperatures of growth (37 °C for bacteria and 28 °C for yeast and fungi), and the optical densities (O.D) of the microbial growth were determined by a spectrophotometer conducted at 600 nm. Experiments were performed in triplicate for each strain of the microorganisms under study, and the results are presented as mean ± standard deviation (SD). The obtained results are the average value. N.B: Inhibition of microbial growth (%) = 100 − [(O.D of sample (trial)/O.D of standard) × 100].

#### Longer-term stability assay

The long-term stability of the prepared glass and glass–ceramic samples was determined through re-assessment of the antimicrobial activity test after approximately 9 months of storage. The samples were stored under the ambient laboratory conditions in sealed sample bags; they were not exposed to direct sunlight. No controlled temperature, humidity or light conditions were used during the storage period. The same antimicrobial testing protocol previously mentioned in this study is applied. The test was performed on the same microbial strains under the same conditions of incubation.

#### Cytotoxicity assessments

**Mammalian cell lines: HFB4** cells** (**Human normal melanocytes cell line) were collected from the American Type Culture Collection (ATCC, Rockville, MD).

**Chemicals Used:** Dimethyl sulfoxide (DMSO), trypan blue dye, and MTT were purchased from Sigma (St. Louis, Mo., USA). Fetal Bovine serum, HEPES buffer solution, DMEM, L-glutamine, 0.25% Trypsin–EDTA, and gentamycin were purchased from Lonza (Belgium).

**Cell line Propagation:** The cells were cultured in Dulbecco’s modified Eagle’s medium (DMEM) enriched with 10% heat-inactivated fetal bovine serum, HEPES buffer, 1% L-glutamine, and 50 µg/ml gentamycin. All cells were kept at 37 °C in a moisturized environment with 5% CO_2_ and were sub-cultured twice a week.

**Viability assay for cytotoxicity evaluation:** For cytotoxicity assay, the cells were plated in a 96-well plate at a cell concentration of 1 × 10^4^ cells per well in 100 µl of growth medium. Freshly made medium loaded with different concentrations of the sample under test was introduced after 24 h of cell plating. The tested compound was serially diluted (two-fold) and applied to confluent monolayers of cells seeded into 96-well, flat-bottomed microtiter plates (Falcon, NJ, USA) using a multichannel pipette. The microtiter plates were incubated at 37 °C in an incubator with controlled humidity with 5% CO_2_ for 24 h. Three wells were used for each concentration of the test sample. Control cells were incubated without a test sample and with or without DMSO. The small amount of DMSO in the wells (maximal 0.1%) was not affect on the experiment. After the incubation of cells, the yield of the viable cells was measured by the colorimetric method. After 24 h of incubation, the MTT test was used to determine the number of viable cells. In brief, the media was eliminated from the 96-well plate and replaced with 100 µl of fresh culture RPMI 1640 medium without phenol red, then 10 µl of the 12 mM MTT stock solution (5 mg of MTT in 1 mL of PBS) to each well, including the untreated controls. The 96-well plates were then incubated at 37 °C and 5% CO_2_ for 4 h. An aliquot of the media (85 µl) was removed from the wells, and 50 µl of DMSO was added to each well and completely mixed with the pipette and incubated at 37 °C for 10 min. Then, the optical density was measured at 590 nm with the microplate reader (Sunrise, TECAN, Inc., USA) to determine the number of viable cells, and the percentage of viability was calculated using Eq. 3

3 $$\left[ {\left( {{\text{ODt}}/{\text{ODc}}} \right)} \right] \, \times \, 100\%$$

where ODt and ODc are the mean optical density of wells treated and untreated with the tested samples, respectively. The relationship between sample concentrations and surviving cells is represented graphically to get the survival curve of each cell line after treatment with the tested samples. The Cytotoxic Concentration (CC_50_), the sample concentration that can induce cytotoxic effects in fifty percent (50%) of the intact cells, was measured from graphic plots of the dose response curve for each conc. Using Graphpad Prism software (San Diego, CA., USA).

## Results and discussion

### X-ray diffraction patterns

**Fig. 1:**
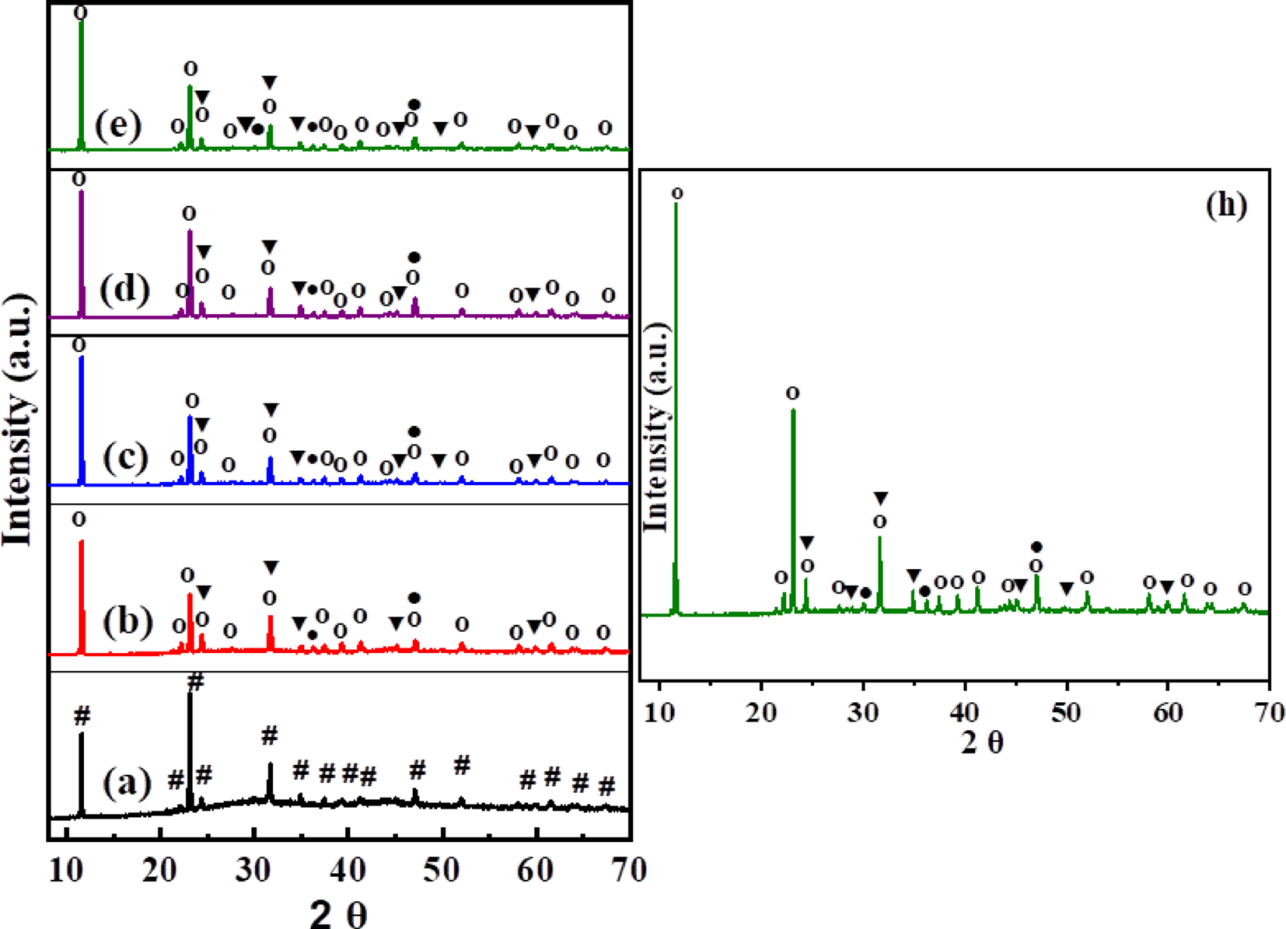
XRD pattern of glass–ceramics (**a**) BGCZr0, (**b**) BGCZr1, (**c**) BGCZr2, (**d**) BGCZr3, and (**e**) BGCZr4 where # β-CaAl_2_B_2_O_7_, ○ α-CaAl_2_B_2_O_7_, ▼ Nano-sized ZrO_2_, ● CaB_2_O_4_. (**h**) Enlarged view of the individual sample pattern extracted from (**e**), showing the actual peak intensities more clearly.

Figure 1 displays XRD patterns of the crystallized samples (glass–ceramics) BGCZr0, BGCZr1, BGCZr2, BGCZr3, and BGCZr4. As shown, certain crystalline phases were separated through heat treatment of the parent glass samples. Initially, the β-CaAl_2_B_2_O_7_ phase (card no. 00-019-0206) was formed in the base sample (BGCZr0). Upon the introduction of ZrO_2_, the β-CaAl_2_B_2_O_7_ phase transformed into the α-CaAl_2_B_2_O_7_ phase (card no. 00-019-0205). In addition to this transformation, two phases, which are ZrO_2_ (card no. 00-065-0687) and CaB_2_O_4_ (card no. 01-075-0640), were also observed. The crystallite size of the formed crystalline ZrO_2_ phase was calculated using the Scherrer Eq. (4)^50,51^.

4 $${\text{d}} = {\rm K}\lambda /{\text{B}}\cos \theta$$

where d is the crystallite size, K is the Scherrer constant (0.9), λ is the wavelength of the X-ray (0.15406 nm), B is the FWHM of the peak, and θ is the Bragg angle. The calculated crystallite sizes of the nano-ZrO₂ phase for each sample are as follows: (43–79 nm), (48–79 nm), (24–79 nm), and (23–79 nm) for BGZr_1_, BGZr_2_, BGZr_3_, and BGZr_4_, respectively, indicating the formation of nanocrystalline ZrO_2_ embedded within the glassy matrix^52^. The relatively broad range of crystallite sizes reflects the wide size distribution of the nano-ZrO₂ phase within each sample. Addition of ZrO_2_ increases the crystallinity of the prepared glass–ceramics related to its performance as a nucleating agent^53^. As shown, XRD peaks appeared relatively sharp with a small crystallite size, although, the sharpness of the XRD peaks often suggests larger crystallites due to the Scherrer effect. However, peak sharpness alone is not a definitive indicator of crystallite size, as it can also depend on instrumental resolution, strain effects, or the degree of crystallinity^54,55^. A similar XRD profile with relatively sharp peaks was reported for ZrO₂ nanoparticles with crystallite sizes of 12–16 nm^56^. Also, Bahammam et al. in their study of preparation of ZrO₂ nanoparticles reported a crystal size of 40–75 nm while all diffraction peaks appear to be significantly sharp, and they attributed this behavior to the purity and crystallinity of the prepared zirconium oxide^43^. Well-controlled crystallization with proper glass matrix composition and heat treatment leads to small, homogeneously distributed crystals that can reduce residual stresses. Several studies have reported the formation of ZrO₂ nanocrystals embedded in glass or glass–ceramic matrices, and in some of these, crystallite sizes in the nanometer range were obtained using XRD. For example, in a fluorosilicate glass–ceramic system, the average crystallite size of zirconia was calculated by XRD to decrease from ~ 27 to ~ 17 nm with increasing ZrO₂ content, and the XRD profile exhibited relatively sharp peaks^57^. This demonstrates that the presence of a glass matrix does not necessarily prevent sharp diffraction peaks or the determination of relatively small crystallite sizes.

### FTIR measurements

**Fig. 2:**
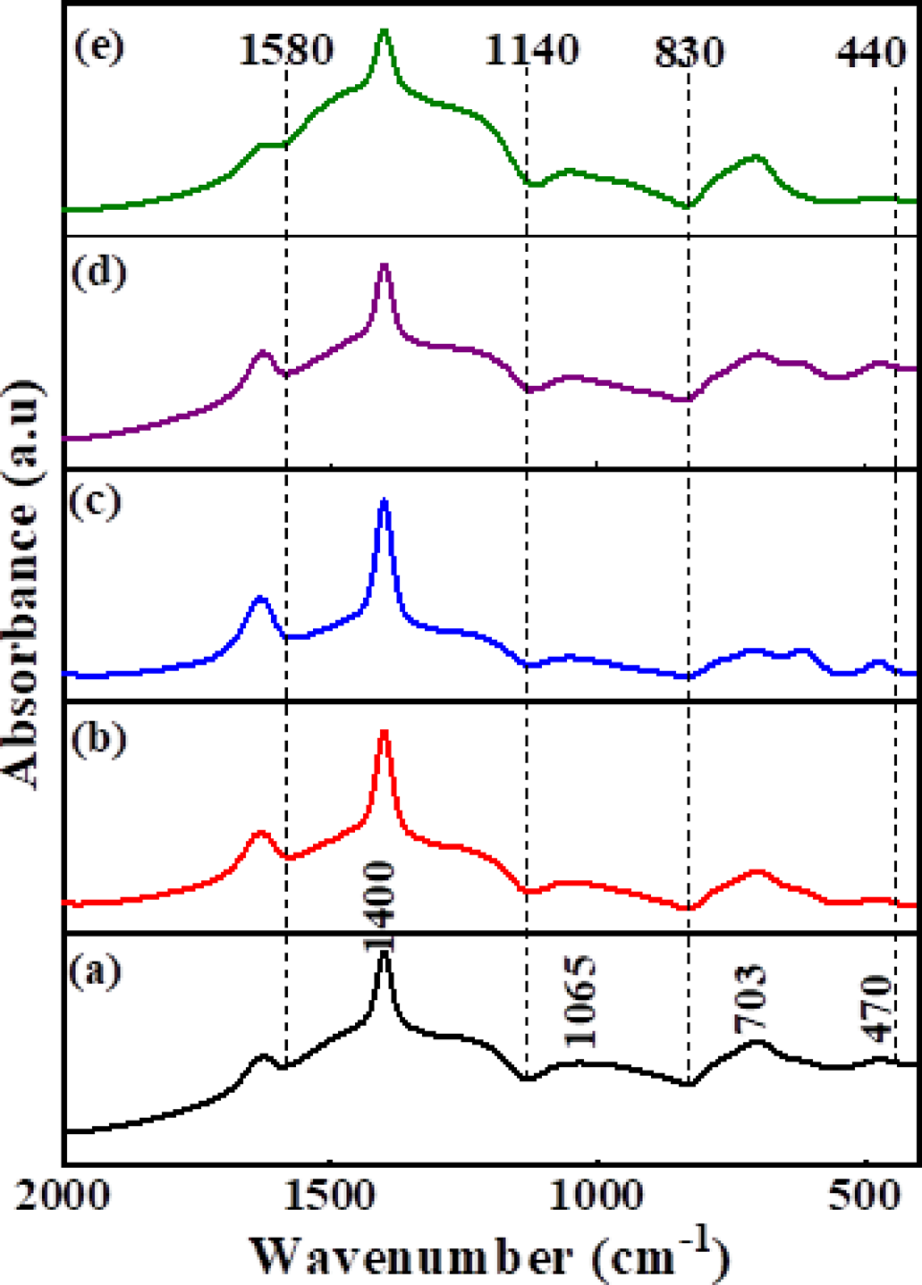
FTIR absorption spectra of base and ZrO_2_-doped borate glass samples where (**a**) BGZr0, (**b**) BGZr1, (**c**) BGZr2, (**d**) BGZr3, (**e**) BGZr4.

Figure 2 exhibits the FTIR spectra of the base and ZrO_2_-doped borate glass samples. As expected in borate-based glasses, the FTIR spectra show three main broad absorbance bands related to the stretching and bending vibrations of triangular and tetrahedral borate groups in their specific vibrational regions ^58–61^ as follows:

1. The broad band occurred within the range (1600–1200 cm^-1^) can be ascribed to the stretching vibrations of B–O bonds from triangular borate units (BO_3_).
2. The vibrational band located in the range (1200–800 cm^-1^) is related to symmetric and asymmetric stretching of B–O in tetrahedral borate groups (BO_4_).
3. The bands observed in the range of (800–600 cm^-1^) can be assigned to the bending modes of B–O–B of (BO_3_ and BO_4_) units.
4. The far infrared region (550–400 cm^-1^) is correlated to cation vibrations of Ca^2+^, Al^3+^, and Zr^4+^ units^62^.

**Fig. 3:**
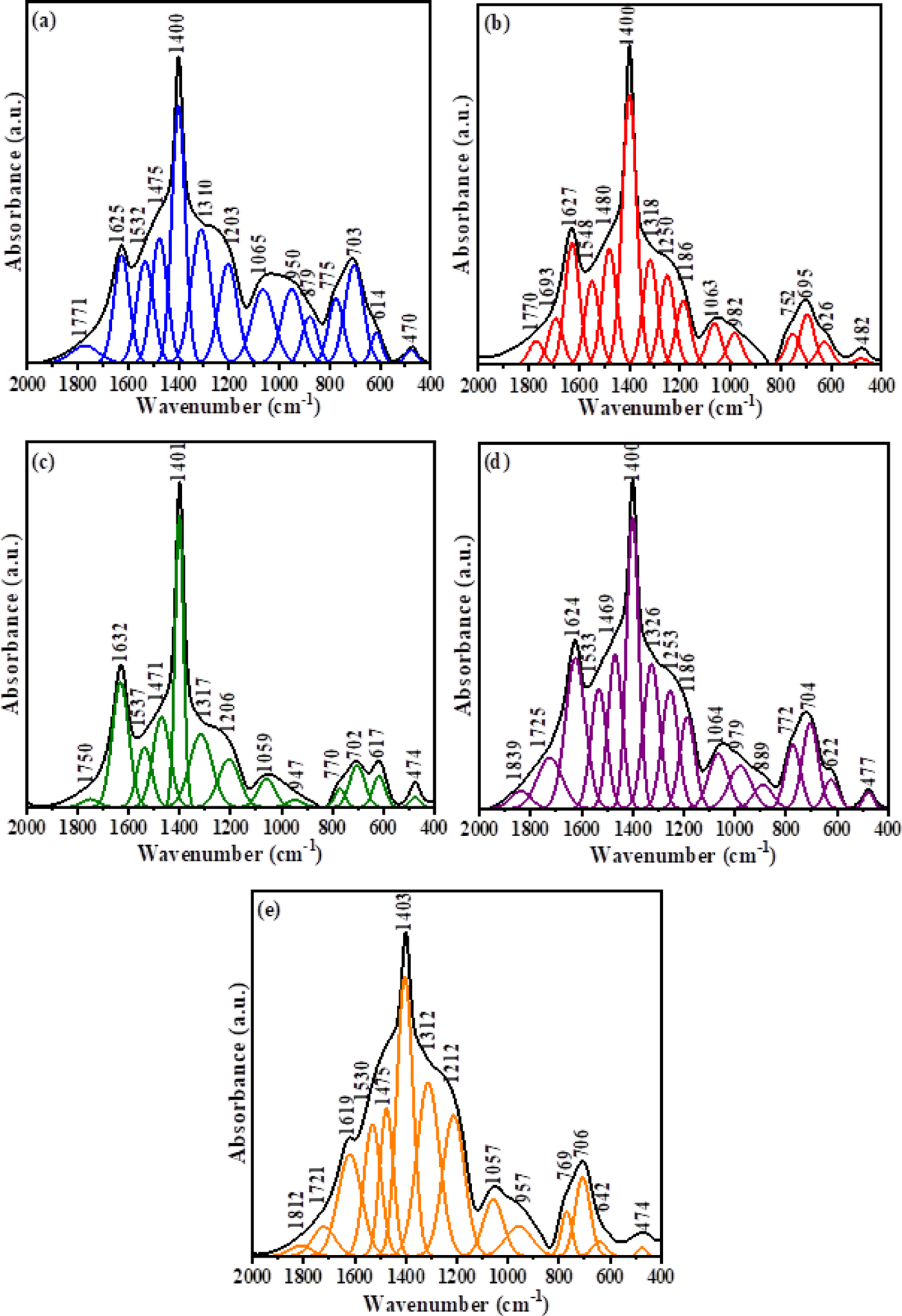
Deconvoluted FTIR spectra of glass samples (**a**) BGZr0, (**b**) BGZr1, (**c**) BGZr2, (**d**) BGZr3, (**e**) BGZr4.

Figure 3 shows the deconvoluted FTIR spectra of the prepared glass samples. The FTIR spectra were deconvoluted using Peak Fit program: SeaSolve software. Inc. USA. Deconvolution is important for obtaining an explanation about the overlapping bands distribution, identification of distinct vibrational modes, and the detection of changes in network structure upon ZrO₂ incorporation. As observed, the vibrational band intensities of triangular borate groups (BO_3_) at (1600–1200 cm^−1^) are higher than those of tetrahedral borate groups (BO_4_) at (1200–800 cm^−1^). This behaviour can be accounted for the presence of alkali (LiF) and alkaline earth oxides (CaO) in the glass matrix, which convert some of the borons from triangular to tetrahedral coordination until a critical concentration of tetrahedrally coordinated borons. Beyond this concentration, the oxides act as modifiers, leading to the formation of non-bridging oxygen as reported in^62,63^. It is noticed that the main FTIR characteristic broad bands of the base sample (BGZr_0_) and their corresponding ZrO_2_-doped borate glass (BGZr_1_, BGZr_2_, BGZr_3_, and BGZr_4_) are similar, with some changes in positions of certain peaks towards higher or lower wavenumber upon the insertion of ZrO_2_ into the glass matrix. The peak located at 470 cm^−1^ related to the metal cations vibrations (Ca^2+^)^64^ shifts to a higher wavenumber (482–474 cm^−1^), and this may be due to the additional vibrations of Zr^4+^ ions. The vibrational band at (614 cm^−1^), which appeared due to the bending vibrations of B–O–B, shifts to a higher wave number (617–642 cm^−1^) upon the gradual addition of ZrO_2_. The band observed at (695–706 cm^−1^) is due to the bending vibration of B–O–B in BO_3_ groups and the presence of Zr–O–Zr vibrations or ZrO_4_^65^. The assignments of the vibrational bands are listed in Table 2. N_4_ is the fraction that represents the ratio of ∑ the area of peaks related to BO_4_ structural group units to ∑ the area of peaks related to BO_3_ and BO_4_ structural group units, and can be calculated depending on deconvolution results^66,67^ by Eq. 5

5 $${\text{N}}_{4} = {\text{A}}_{4} /\left( {{\text{A}}_{3} + {\text{A}}_{4} } \right)$$

where A_3_ and A_4_ are ∑ the areas of peaks related to BO_3_ and BO_4_ structural borate units, respectively. The values of A_3_, A_4_ and calculated N_4_ are listed in Table 3

**Table 2:** The assignments of the vibrational bands ^64,65,69,71^.

Peak no.	Wavenumber (cm^-1^)					Assignments
	BGZr0	BGZr1	BGZr2	BGZr3	BGZr4	
1	470	482	474	477	474	Vibrations of metal cations Ca^2+^, Zr^4+^
2	614	626	617	622	642	B–O–B bending vibrations of BO_3_ units
3	703	695	702	704	706	B–O–B bending vibrations and Zr–O–Zr vibrations of ZrO_4_
4	775	752	770	772	769	bending vibration of B–O–B linkage
5	879	–	–	889	–	BO_4_ stretching of tri-, penta, and diborate groups
6	950	982	947	979	957	
7	1065	1063	1059	1064	1057	
8	1203	1186	1206	1186	1212	Asymmetric stretching vibrations of (BO_3_)
9	–	1250	–	1253	–	
10	1310	1318	1317	1326	1312	
11	1400	1400	1401	1400	1403	
12	1475	1480	1471	1469	1475	
13	1532	1548	1537	1533	1530	
14	1625	1627	1632	1624	1619	Vibrations of water, OH groups
15	1771	1770	1750	1725	1721	
16	1874	–	1835	1839	1812

**Table 3:** Area of BO_3_ groups (A_3_), area of BO_4_ groups (A_4_), fraction of tetrahedral coordinated boron atoms (N_4_) values.

Glass code	Area of BO_3_ units (A_3_)	Area of BO_4_ units (A_4_)	N_4_
BGZr0	159.698	53.173	0.249
BGZr1	122.849	12.371	0.091
BGZr2	81.053	6.483	0.074
BGZr3	152.171	28.389	0.157
BGZr4	232.768	28.231	0.108

It is observed that there is a decrease in the value of calculated N_4_ by the addition of ZrO_2_ into the glass matrix, indicating a decrease of the number of BO_4_ groups. The presence of zirconium ions in the glass structure induces the BO_4_ → BO_3_ back conversion, resulting in an increase of non-bridging oxygen (NBO_S_). Accordingly, one can conclude that ZrO_2_ acts as a network modifier in the glass system under study^68,69^. The values of N₄ with ZrO₂ content do not follow a specific trend. First, the decrease in N₄ for low ZrO₂ content suggests that Zr^4+^ acts mainly as a network modifier, generating more non-bridging oxygens. Then, the slight N₄ value increase for BGZr_3_ may be due to the partial incorporation of Zr^4+^ into the network, which can temporarily stabilize BO₄ units. For further addition (BGZr_4_), the modifier effect becomes dominant again, leading to a small decrease in N₄. In this study, the investigated range of ZrO₂ content may not be sufficient to show the network-forming effect of ZrO₂, and the observed changes in N₄ remain relatively limited. Overall, the N₄ values remain lower than the base glass, indicating that ZrO₂ acts as a modifier in this concentration range.

**Fig. 4:**
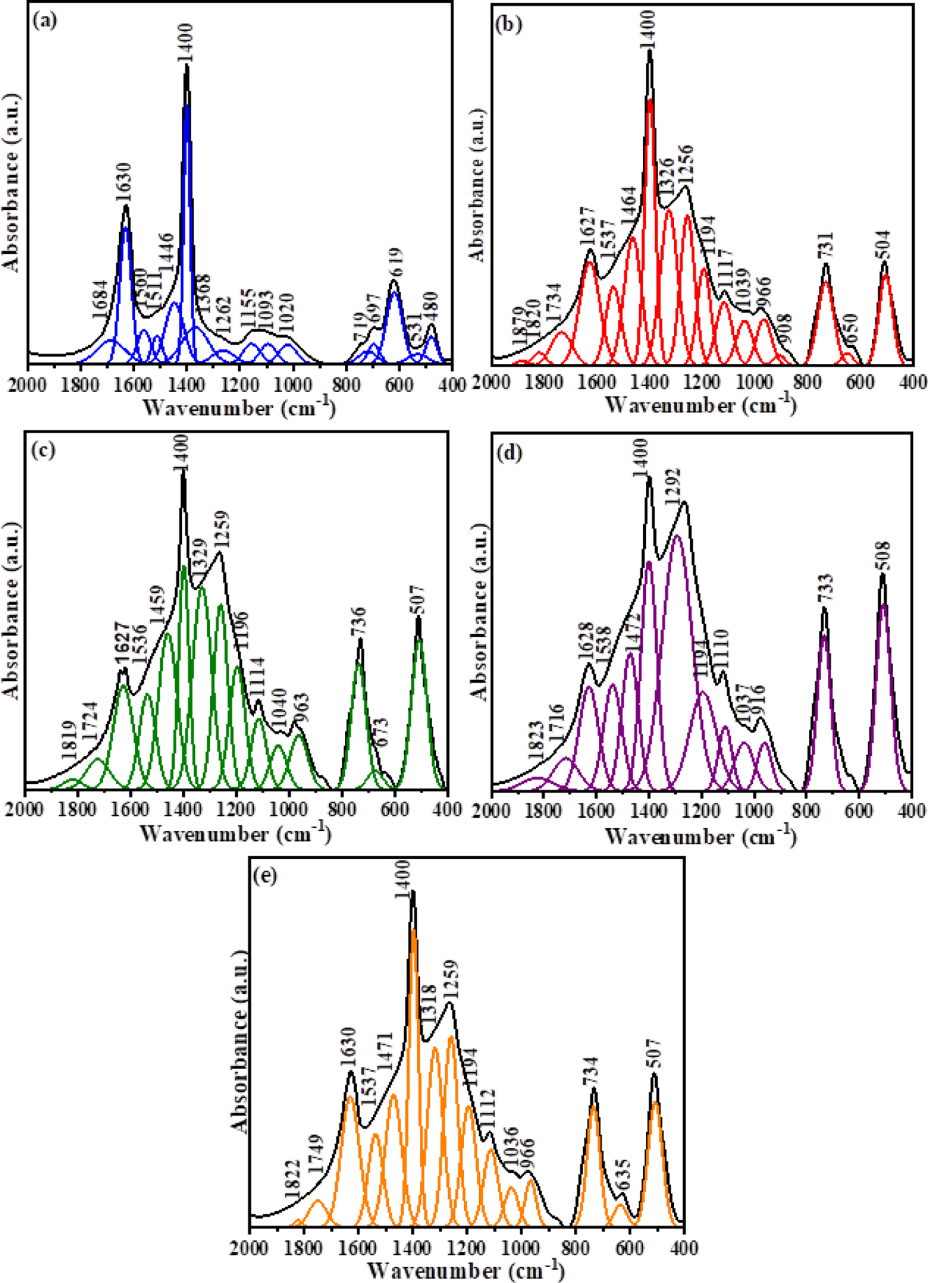
Deconvoluted FTIR spectra of glass–ceramics (**a**) BGCZr0, (**b**) BGCZr1, (**c**) BGCZr2, (**d**) BGCZr3, (**e**) BGCZr4.

Figure 4 displays the FTIR of glass–ceramic samples. The FTIR exhibits the same characteristic main spectral bands of their parent glasses, but these vibrational bands are observed to split into distinct sharper peaks related to the fine crystalline derivatives of BO_3_ and BO_4_^70^. The crystallization in glass means converting the random structure of glass to rearranged structure, which making the structure more stable and has a low activation energy. This can be identified by the formation of new phases moving in the residual glassy phase.

### Density measurements

**Fig. 5:**
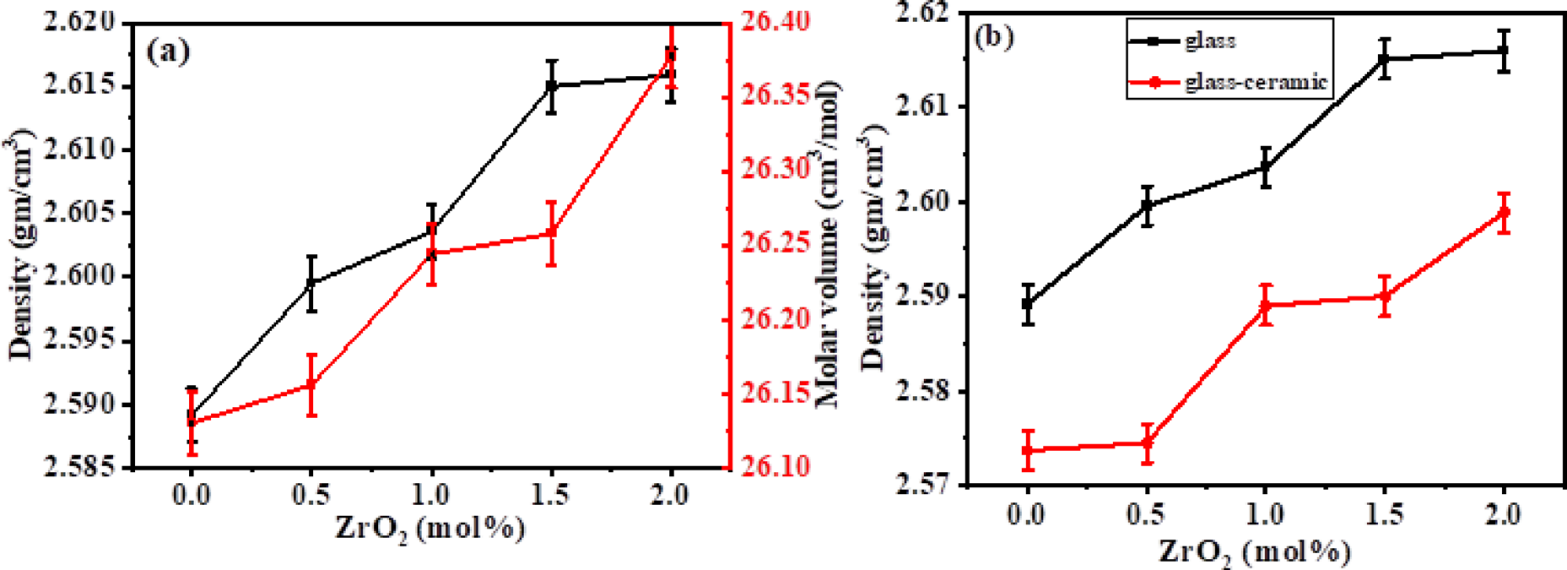
Density and Molar Volume of glass (**a**) and Density of glass and glass–ceramics (**b**). Error bars correspond to a relative uncertainty of ± 0.08%, applied for visual clarity, while the experimental precision was ± 0.02 g/cm^3^.

Density is considered a helpful parameter for giving information about the glass structure behaviour. The density relies on the compactness, the coordination number, the cross-link densities, the geometrical configurations, and dimensions of the interstitial spaces of the glass^72^. Figure 5(a) displays the variation of the measured density and the calculated molar volume as a function of ZrO_2_ content in the glass matrix. As displayed, the density of the glass rises from 2.5892 to 2.6159 g/cm^3^ by the substitution of CaO by ZrO_2_. The gradual increase of density upon the inclusion of ZrO_2_ into the glass matrix at the expense of CaO was attributed to the heavier molecular weight of ZrO_2_ (123.222 g/mol) than CaO (56.078 g/mol)^65^. In general, the behaviour of density and molar volume is opposite, while in this study, they have the same trend. Increasing the molar volume by increasing ZrO_2_ content in this glass system from 26.1305 to 26.3775 cm^3^/mol may be accounted for the expansion of the network as a result of increasing the number of non-bridging oxygen (NBOs) upon the BO_4_ → BO_3_ back conversion, as confirmed through FTIR study. The increase in the molar volume indicates the presence of ZrO_2_ as a modifier in the glass matrix by occupying the interstitial space in the network and generating NBOs inside the structure^68^. Generally, density decreases with an increase in NBOs, however it seems opposite in this case due to the huge variation in molecular masses between CaO (56.078 g/mol) and ZrO_2_ (123.222 g/mol), that has more effect on density than the effect of the formation of NBOs. As observed in Fig. 5(b), crystallization may reduce the density of the formed glass ceramics compared to their parent glasses; however, this is not always the case. The reduced density in glass–ceramics may occur when the formed crystalline phases are less dense than the parent glass^73^ or when crystallization introduces microstructural changes (such as porosity, fracture, and voids)^74^ leading to an overall decrease in density. Furthermore, the addition of nucleating agents (such as ZrO_2_) often leads to a decrease in the overall density and the activation energy of the system, which eases the processing of glasses into glass–ceramics^73,75^. Table 4 displays the values of measured density, molar mass, and calculated molar volume of glass and glass ceramics.

**Table 4:** Density, molar mass and molar volume of glass and glass–ceramics.

Samples code		Density (g/cm^3^) (± 0.02)	Molar mass (g/mol)	Molar volume (cm^3^/mol)	Samples code		Density (g/cm^3^) (± 0.02)
Glass	BGZr0	2.5892	67.6572	26.1305	Glass–ceramics	BGCZr0	2.5737
	BGZr1	2.5995	67.9929	26.1561		BGCZr1	2.5745
	BGZr2	2.6036	68.3287	26.2439		BGCZr2	2.5890
	BGZr3	2.6150	68.6644	26.2578		BGCZr3	2.5900
	BGZr4	2.6159	69.0001	26.3775		BGCZr4	2.5988

### Field emission scanning electron microscope (FE-SEM) images

**Fig. 6:**
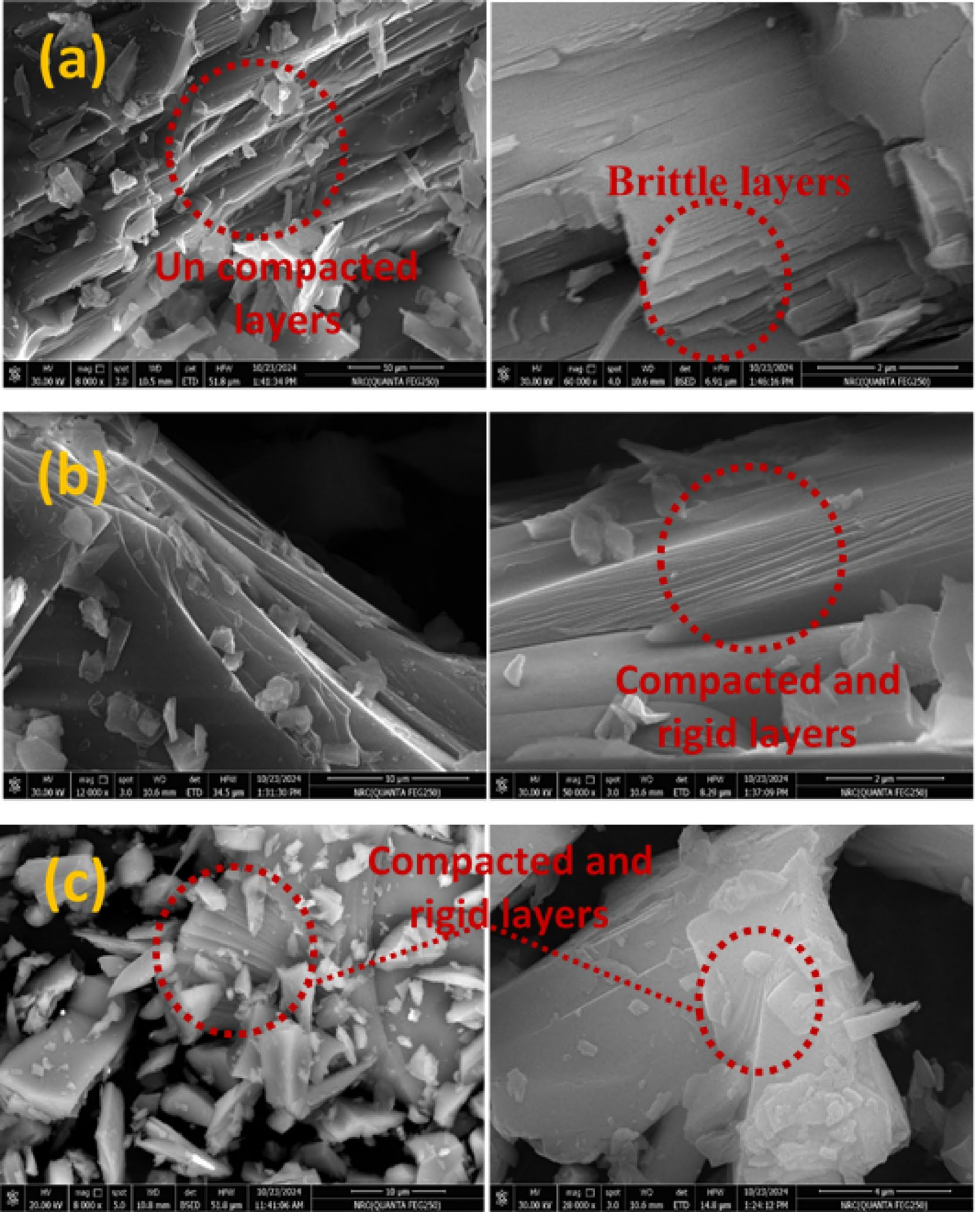
FE-SEM images of (**a**) BGCZr0, (**b**) BGCZr2 and (**c**) BGCZr4.

Figure 6 displays FE-SEM images of the glass–ceramic samples BGCZr0, BGCZr2, and BGCZr4. It is shown that the images display crystalline structure (as confirmed by XRD patterns) that comprises a group of sheets (layers). For BGCZr0, these layers are uncompacted and brittle, while the substitution of CaO by ZrO_2_ increases the compactness and rigidity of layers (BGCZr2 and BGCZr4). This can be rationalized by the great effect of ZrO_2_ on the crystallization process, where ZrO_2_ acts as a nucleating agent.

### Dielectric study

The dielectric properties of glass and glass–ceramics are essential in assessing their appropriateness for many electrical and energy storage applications. They are affected by various factors, including composition, microstructure, and heat treatment. The transition from an amorphous glassy phase to a partly crystalline glass–ceramic structure frequently results in substantial alterations in dielectric properties, chiefly attributable to variations in ionic mobility, interfacial polarization, and defect concentrations.

In this section, the dielectric properties of the synthesized glass and glass–ceramic samples were investigated over a wide frequency range at 30 °C. Essential properties like permittivity, loss tangent, and AC conductivity, as well as real and imaginary parts of electric modulus, are examined to elucidate the fundamental conduction mechanisms and polarization effects. Furthermore, the influence of phase transitions and crystalline phase distribution on electrical performance is evaluated, offering insights for the improvement of these materials in technological applications.

#### For glass samples

**Fig. 7:**
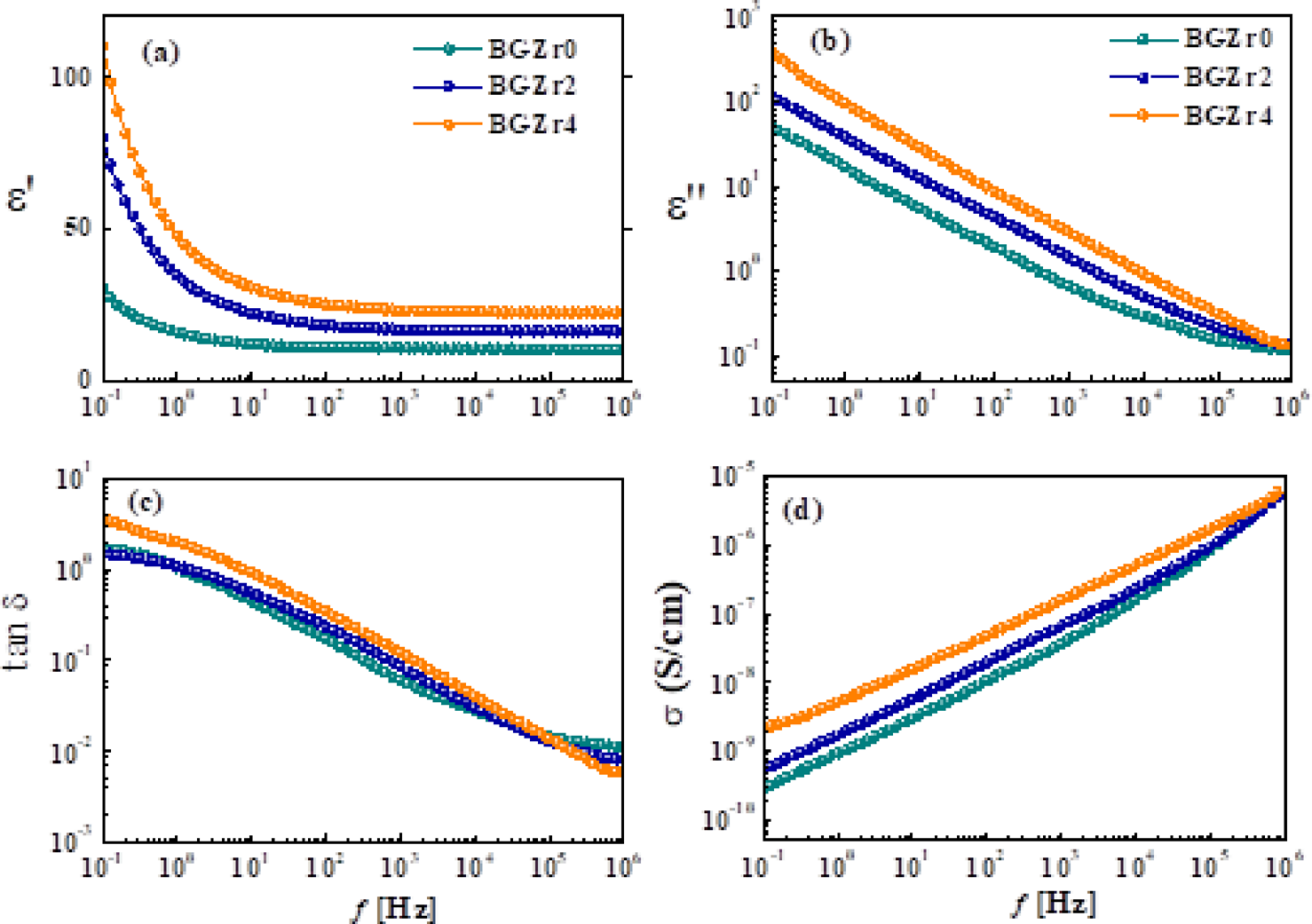
The frequency dependence of permittivity, ε′ (**a**), the dielectric loss, ε″ (**b**), the loss tangent, tanδ (**c**), and AC conductivity, σ (**d**) for BGZr0, BGZr2, and BGZr4 glass samples measured at 30 °C.

Figure 7(a–c) illustrates the variation of permittivity (ε′), dielectric loss (ε″), loss tangent (tanδ), and AC conductivity (σ_ac_) within the frequency range (0.1 Hz-1 MHz) at 30 °C for BGZr0, BGZr_2_, and BGZr_4_. Furthermore, for ease of comparison, the dielectric properties selected at constant frequency values (1Hz, 1 MHz) are summarized in Table 5. ε′, ε″, and tanδ have high values at low frequencies, which generally decrease with increasing frequency due to a total polarization decrease. In particular, ε′ decreases faster with increasing frequency up to 100 Hz, then becomes almost constant at higher frequencies, indicating that two mechanisms dominate the dielectric properties of the samples.

**Table 5:** dielectric properties (ε′, ε″, tanδ, and σ) and HN-fitting parameters (τ, α, and β) for glass and glass–ceramic samples.

Sample code	Glass samples									
	ε′		tanδ		ε″		σ (S/cm)	HN parameters		
	1Hz	1MHz	1Hz	1MHz	1Hz	1MHz		τ (sec)	α	β
BGZr0	16.03	10.26	1.04	0.0120	16.75	0.117	2.73E-10	0.0428	0.42	0.94
BGZr2	34.72	16.29	1.11	0.0083	37.59	0.135	1.91E-9	0.0350	0.46	0.88
BGZr4	47.91	22.47	1.98	0.0059	44.97	0.133	6.65E-9	0.0078	0.43	0.90
*Glass–ceramic samples*										
BGCZr0	32.32	20.51	0.47	0.0048	13.95	0.098	6.87E-10	0.132	0.43	0.87
BGCZr2	49.60	23.26	0.63	0.0049	31.33	0.112	2.19 E-9	0.0358	0.44	0.84
BGCZr4	52.44	23.11	0.17	0.0048	8.95	0.112	9.21E-9	0.0052	0.49	0.72

The frequency-dependent dielectric properties have been demonstrated by evaluating the permittivity (ε′) behaviour of a glass sample BGZr_4_ at 1 Hz and 1 MHz as a representative example. Here, ε′ is ~ 15–48 at 1 Hz, which then decreases with frequency and attains its minimum value, i.e. 10 ~ 22 at 1 MHz. This seems to be in agreement with the data reported for borate-based and bioactive glass systems such as CaO–B_2_O_3_–SiO_2_ and TiO_2_- or ZnO-doped borate glasses, where ε′ is ~ 15–60 at low frequencies^76–80^.

The coexistence of space charge polarization and direct current or dc conductivity (σ_dc_) could be responsible for the high values seen in permittivity at low frequencies, while at higher frequencies, only intrinsic electronic and ionic polarization dominated, resulting in a decrease in permittivity. Space charge or Maxwell Wagner Sillar (MWS) polarization, arising from the building up of charges at interfaces of different regions within the disordered structure, like glass, and appears in heterogeneous materials, due to the differences in conductivities and permittivities of phases contained in samples^76^. On the other side, DC conductivity (σ_dc_) arises from the random fluctuation motions of free charge carriers whose values are much lower than those of AC conductivity (σ_ac_).

For being a glass-disordered structure, the electric dipole alignment diminishes, leading to increased energy losses, as indicated by increased losses in Fig. 7(b, c)^77^. On the other hand, AC conductivity (σ) shown in Fig. 7(d) increases with increasing frequency, following a power-law relationship with frequency, aligning with the universal dielectric response. This signifies hopping conduction of charge carriers between localized states. Such a conductivity increase is in good agreement with short-range hopping conduction typical for disordered systems^78^.

The observed increase in ε′ with the addition of ZrO₂ suggests that ZrO₂ contributes to greater polarizability in the glass network, making the glass better at storing electrical energy. For such a case, ZrO₂ either introduces more non-bridging oxygens (NBOs), modifying the glass network structure, or enhances local polarizable units, allowing greater space charge and dipole polarization, especially at low frequencies^79^.

The increase in ε″ with ZrO_2_ addition means more energy is dissipated in the present samples. This is because ZrO_2_ probably allows more ions, causing a conduction loss increase or dipole reorientation contributing to loss mechanisms. This effect is more obvious at low frequencies. At higher frequencies, dipoles and space charges cannot follow up the fast changes in the electric field, so the loss drops off. The increase in loss energy relative to storage energy (tanδ) with ZrO_2_ addition means more energy is lost due to higher ionic or dipolar contributions. This agreed with the data reported previously for Zr-doped glasses^44^.

AC conductivity (Fig. 7d) of the present samples shows an increase with the frequency increase following Jonscher’s power law (σ & ω^n^, 0 < n < 1), typical of hoping conduction in disordered systems. Its values (~ 10^–9^–10^–5^ S/cm) falls within the range typical ionic conducting borate glass–ceramics (~ 10^–9^–10^–6^ S/cm), confirming that incorporation of ZrO_2_ enhancing polarizability and charge carrier hoping.

ZrO_2_ creates sites and pathways that promote ion transport via the hoping mechanism. The presence of zirconium ions (Zr^4+^) in the glass structure induces the BO_4_ → BO_3_ back conversion, resulting in an increase of non-bridging oxygen (NBOs). This confirms that hopping conduction dominates the charge transport mechanism in both glass and glass–ceramic samples. Furthermore, modifiers like CaO and LiF break boron–oxygen bridges, increasing the proportion of NBOs, thus affecting electrical and structural properties^44^. In conclusion, the findings indicate that modifying the glass composition substantially enhances its dielectric and electrical properties. The substitution of CaO by ZrO_2_ increases AC conductivity while reducing the dielectric losses.

#### For glass ceramic samples

**Fig. 8:**
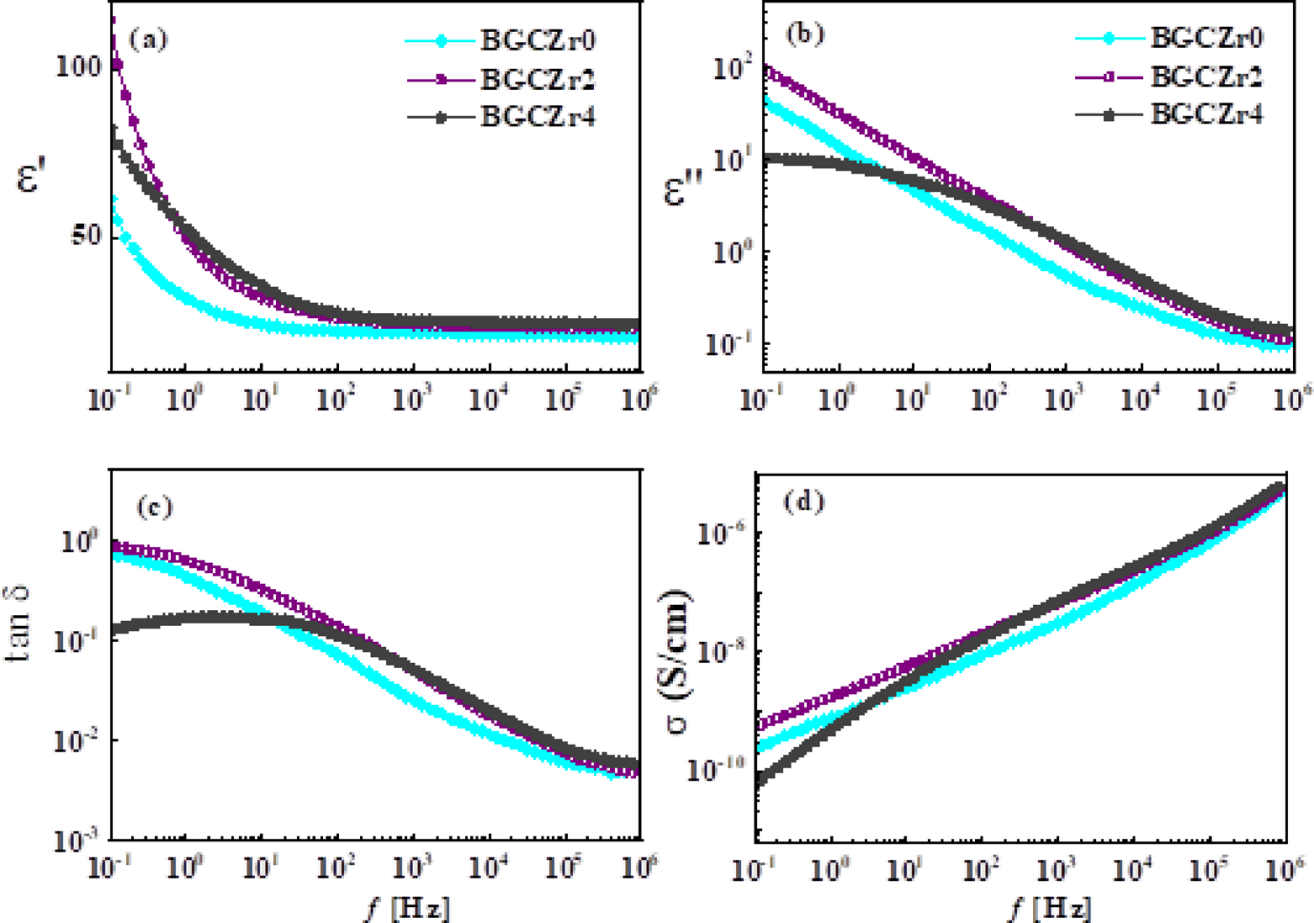
The frequency dependence of permittivity, ε′ (**a**), dielectric loss, ε″ (**b**), loss tangent, tanδ (**c**), and AC conductivity, σ (**d**) for BGCZr0, BGCZr2, and BGCZr4 glass–ceramic samples measured at 30 °C.

Figure 8(a–c) illustrates the permittivity (ε′), dielectric loss (ε″), and loss tangent (tanδ) of BGCZr0, BGCZr2, and BGCZr4 glass–ceramics as a function of frequency range (0.1 Hz–1 MHz) at ~ 30 °C. In comparison with glass samples, the dielectric properties of glass ceramics show similar frequency dependence but relatively higher values due to the partial crystallization of the amorphous glass matrix that leads to structural alteration, affecting the dielectric characteristics. For instance, at 1 MHz, ε′ of glass ceramic sample BGCZr0 is ~ 20, which is two times higher than that of glass sample BGZr0 (~ 10). Zr^4+^ enhances crystallinity as well as dipole and space charge polarizations, hence increasing the permittivity (ε′). This explains why ε′ of Zr^4+^ doped glass ceramics is higher than that of pure glass samples. Furthermore, the dielectric losses (ε′′ and tanδ) of glass ceramics shown in Fig. 8(b, c) display lower values compared to those noticed for glass samples due to the formation of crystalline phases, i.e., α-CaAl_2_B_2_O_7_, Nano-sized ZrO_2_, and CaB_2_O_4_, attributed to diminished defect-related conduction pathways. Thus, these phases make the crystalline structure of glass ceramic stiffer, thereby limiting ion mobility, i.e., lowering dielectric losses and increasing overall permittivity^80^. Based on the above, both crystallinity and Zr^4+^ addition play an essential role in enhancing the dielectric properties of glass ceramic samples to be better than those of glasses.

Usually, the AC conductivity (σ) of a material is affected by several factors such as crystalline phase structure, degree of crystallization, and the presence of dopants. These factors potentially lead to either an enhancement or reduction in conductivity^81,82^. As is clear from Table 5 and Fig. 8(d), the same factors influenced the conductivity of our glass ceramic samples. Here, BGCZr0 shows higher conductivity values (6.87 × 10^–10^ S/cm at 1 MHz) than those of BGZr0 (2.73 × 10^–10^ S/cm at 1 MHz). Also, the conductivity of glass ceramics is affected by Zr^4+^ addition. For instance, the sample of higher Zr^4+^ content (BGCZr0) shows higher conductivity values (9.21 × 10^–9^ S/cm) than BGCZr2 (2.19 × 10^–9^ S/cm) and BGCZr0 (6.87 × 10^–10^ S/cm) due to creating NBOs, sites, and pathways that promote ion transport via a hoping mechanism. In Fig. 8(d), the conductivity of glass ceramics shows a frequency-dependent power-law characteristic, aligning with hopping conduction mechanisms. The crystalline phase did not significantly influence the charge transport channels, demonstrating a slight increase in ionic conductivity due to enhanced structural ordering^83,84^. However, the primary peak of tanδ (Figs. 7d, 8d) is evidently located in the domain where conductivity prevails, indicating the significant role of ionic conduction in dielectric losses^85^. A peak in the dielectric loss spectra arises from space charge or Maxwell Wagner-Sillar (MWS) polarization^76^. As the glass ceramic structure contains crystalline grains embedded in a glass matrix, the major contribution comes from the interfaces between the crystalline and amorphous phases obtained. Furthermore, crystallization introduces heterogeneous conductivity, leading to more pronounced charge accumulation, which in turn increases the interfacial polarization to be much higher than in glass, exploring enhanced dielectric properties^86^. This explains why the glass ceramic samples have better dielectric properties than glass samples. Importantly, the peak broadness in loss tangent spectra indicates the superposition of ionic transport and interfacial polarization mechanisms affecting the dielectric properties of glass ceramic samples at low frequencies. Therefore, electric modulus representation is suggested in this study because it mainly studies relaxation and conduction behaviour, particularly in ionic and disordered systems. Further, it identifies and separates different relaxation processes, especially when they overlap.

**Fig. 9:**
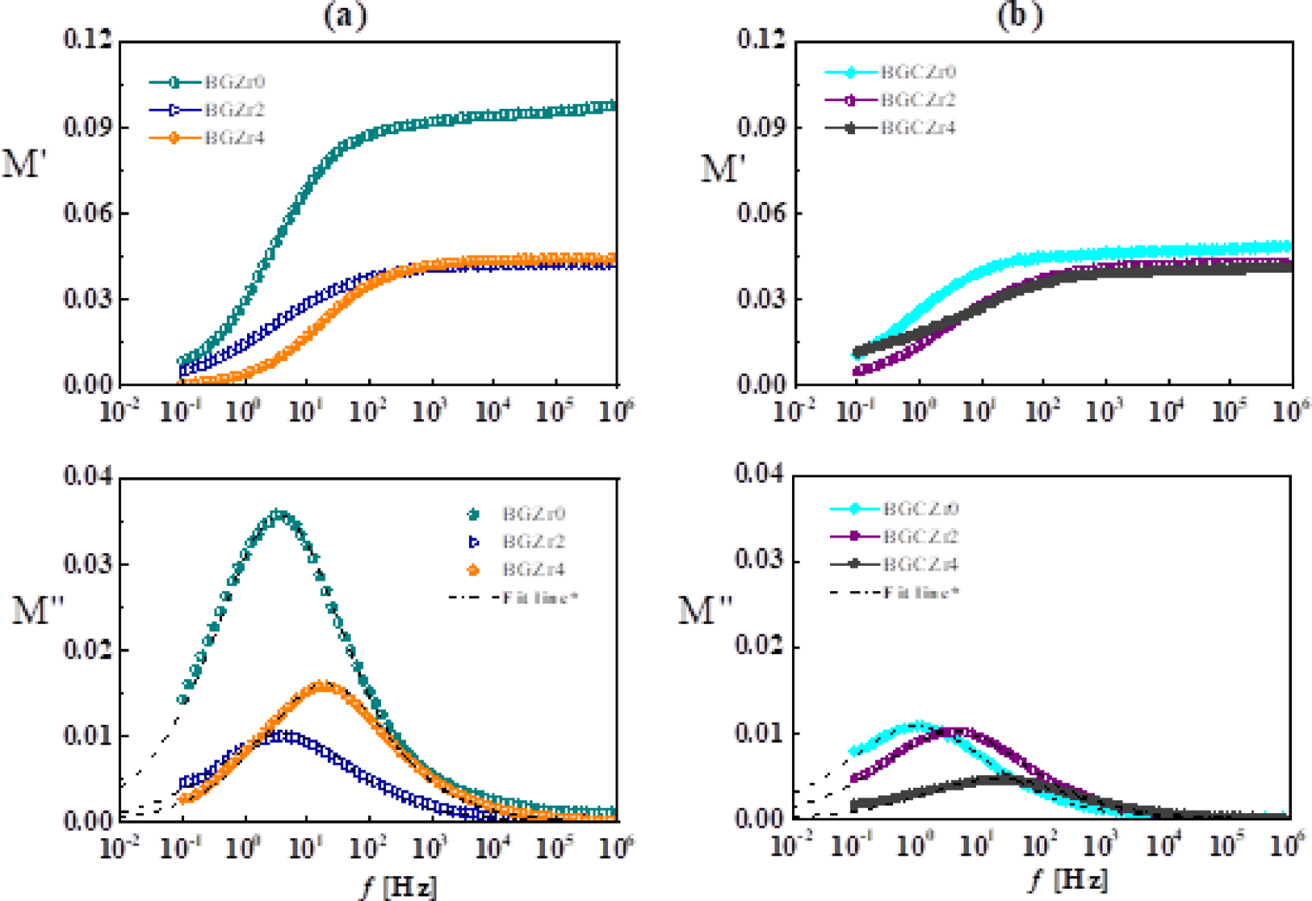
The frequency dependence of real (M′) and imaginary (M′′) part of the complex electric modulus (M*) for glass (**a**) and glass/ceramic samples (**b**) at ~ 30 °C. Fit line in accordance to Havriliak–Negami function.

Figure 9(a, b) illustrates the real (M′) and imaginary (M′′) parts of the complex electric modulus (M*) for glass and glass ceramic samples at approximately ~ 30 °C. M′ starts near zero at low frequencies, showing that long-range movement of charge carriers dominates the dielectric properties at low frequencies. The extended tail observed at lower frequencies indicates a significant capacitance associated with the electrodes, supporting the notion of non-Debye behaviour. Then, M′ increases with increasing frequency and reaches a plateau-like behaviour at high frequencies. This plateau indicates the ability of samples to store electric energy and shows the bulk response. The elevated values of M′ at high frequencies are related to relaxation processes^87^.

The imaginary part of the electric modulus (M″) shows a pronounced peak at low frequencies (I), representing the relaxation process in these materials. The area under this peak denotes a region where charge carriers are constrained to potential wells and can only travel short distances, while the peak position identifies the relaxation time (τ) of ions in the system. The other insignificant peak (II) outside our frequency window in the high-frequency side refers to charge carriers that can go significant distances. The shift in peaks of M″ spectra toward higher frequencies with increasing ZrO_2_ indicates a decrease in relaxation time, consistent with thermally activated short range hoping of charge carriers. A special computer program based on Havriliak-Negami functions is used to fit the data in Fig. 8(a) (M" vs. *f*) plots^88^. The fitting parameters (τ, α, and β) values are included in Table 5. Where, α, and β represent the symmetric and asymmetric peak broadening. These values confirm non- Debye behaviour and show that increasing ZrO_2_ concentration leads to more symmetric relaxation and reduced τ, indicating faster ion dynamics. From the figure, one can notice that the addition of ZrO₂ affects both M′ and M″. ZrO₂ decreases the connectivity in the glass network and slightly decreases the values of M′. It also shifts the M″ peak towards higher frequencies and reduces its height. This suggests that ZrO₂ makes ion movement easier, which speeds up the relaxation process.

The study of M" over all samples within the frequency range reveals one dielectric relaxation, attributed to the conducting grain in the low frequency band. A significant reduction in relaxation time τ_I_ of the relaxation peak (Table 5) is observed when ZrO₂ concentration increases. This is ascribed to the enhancement of conductivity, as illustrated in Figs. 7(d) and 8(d).

#### The correlation between electrical properties and microstructure

From above description, it can be concluded that the formation of distinct crystalline phases (α-CaAl_2_B_2_O_7_, nano-sized ZrO_2_, and CaB_2_O_4_) reported by XRD results (Fig. 1) contributes to increased structural ordering and reduced defect density, suppressing ion mobility and lowers dielectric losses (ε″ and tanδ), as given in Fig. 8(b, c). In addition, the incorporation of Zr^4+^ ions enhance crystallinity and introduces localized sites like NBOs, facilitating space charge and dipolar polarization. This results in enhanced permittivity (ε′), particularly evident in BGCZr4 sample compared to the undoped ones. Zr^4+^ content increases AC conductivity (Fig. 8d and Table 5) due to the formation of hopping pathways and interfacial regions between the residual glassy matrix and crystalline grains. Consequently, interfacial polarization obtained, which significantly contributes to the observed dielectric behavior. The observed broad peaks in tanδ and electric loss modulus spectra further support the presence of overlapping relaxation processes linked to the inhomogeneous microstructure. This means that the interfaces between the crystalline and amorphous phases dominate the relaxation dynamics, reinforcing the role of microstructure in shaping electrical response.

### Biological responses study

#### Antimicrobial activity

**Fig. 10:**
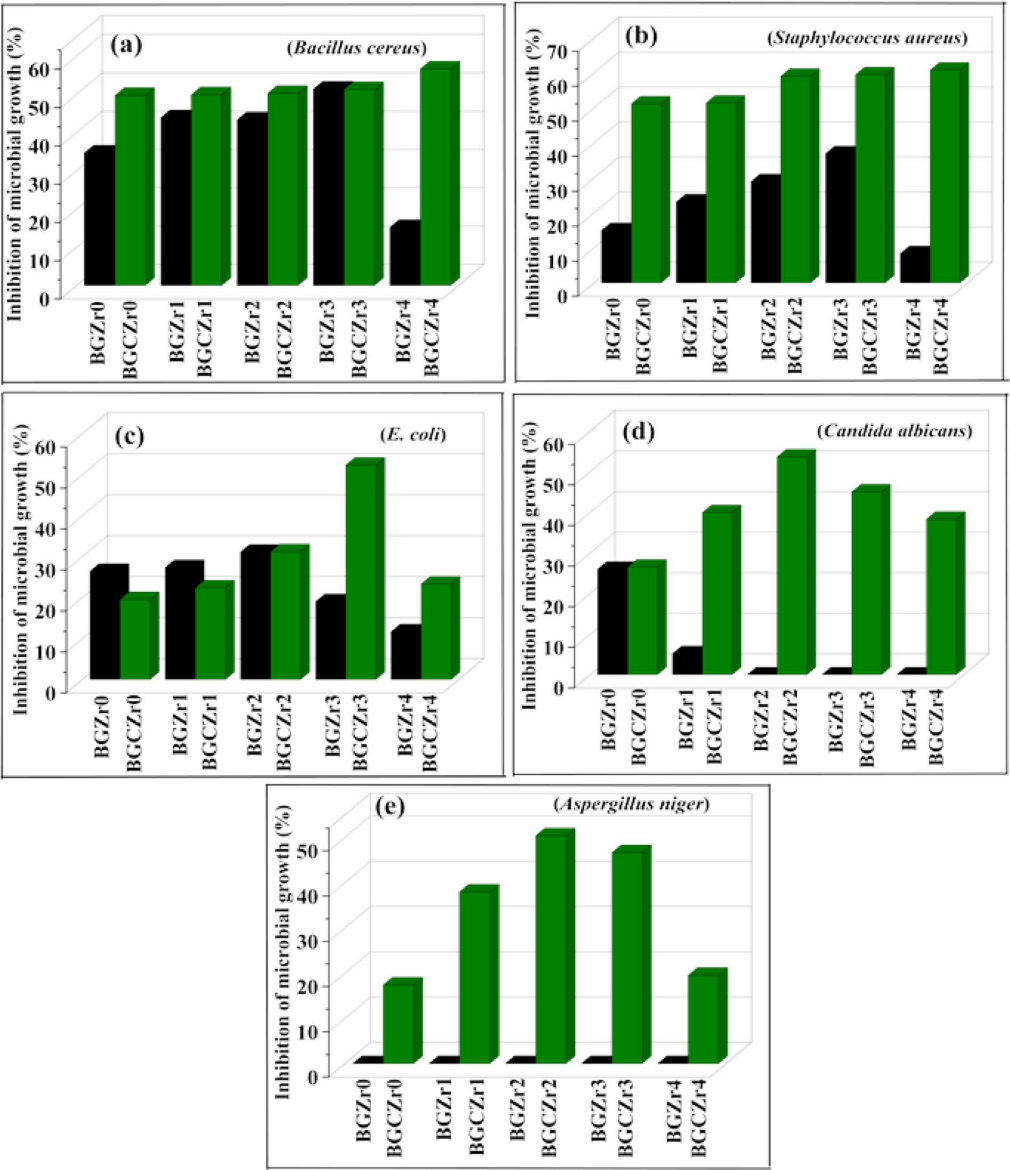
Antimicrobial activity of glass and glass ceramic samples (MIC).

In this study, the antimicrobial results shown in Table 6 and Fig. 10 demonstrated that both glass and glass–ceramics samples exhibit varying antimicrobial responses depending on the concentration of ZrO_2_ content in the glass matrix. As the concentration of ZrO_2_ increased, the antimicrobial effect increased until a definite concentration, and the antimicrobial activity of glass–ceramics is more than that of their parent glasses, which is in agreement with the results reported in many studies^9,89,90^. Boron-containing materials can exhibit antimicrobial activity against several species of bacteria^91,92^ and fungi^93^. Boric acid B(OH)_3_ can be produced by the reaction between boron oxide (B_2_O_3_) in glass composition with H_2_O through a commonly recognized chemical reaction^94^. Boric acid antimicrobial mechanism is attributed to its ability to penetrate bacterial cells via diffusion, resulting in the disruption of the DNA double helix structure, interfering with the integrity of genetic material, and impairing bacterial viability^95,96^. As confirmed by the FTIR study, ZrO_2_ acts as a network modifier that causes an increase in NBO content, leading to reduced glass network connectivity. This poor network connectivity allows the release of alkali metals (such as Ca^2+^) into the surrounding environments, causing an increase in pH and osmotic pressure, which represents an unsuitable environment for bacterial growth and metabolism and causes changes in cell shape, size, and membrane tension, which in turn promotes microbial death^39,97^. Moghanian et al.^98^ studied the antibacterial activity of 58S glass of composition (60 SiO_2_–4 P_2_O_5_–(36-X) CaO–X ZrO_2_) mol% where (X = 0, 5, and 10) mol%. They found that BG-5Zr has higher activity against *S. aureus* than BG-0Zr and BG-10Zr. Also, Hammami et al.^97^ prepared ZrO_2_-modified 45S5 bioglass as a biomaterial for dental implants. Additionally, they studied their antibacterial effect against Gram-positive and Gram-negative bacteria. They found antibacterial activity of the prepared glass samples. However, they noticed that, upon exceeding the ZrO_2_ concentration above 2 mol%, the antibacterial efficacy of the bioglass decreased. They accounted for this decrease by the structural changes of bioglass from amorphous to crystalline with the increase of ZrO_2_. The increase of ZrO_2_ content simulates crystallization of bioglass and hence reduces their solubility, resulting in a reduction in concentration of released ions (Ca^2+^ and Zr^4+^) responsible for increasing pH of the medium. However, in this study, the enhanced antimicrobial activity of glass–ceramics compared to their parent glasses by increasing ZrO_2_ content may be related to the separation of nanocrystalline ZrO_2_ phase (as confirmed through XRD). Nano zirconia is a biologically active material due to its unique properties at the nanoscale, such as, high surface area and increased reactivity compared to their bulk form. The antimicrobial activity of Nano zirconia can be explained through several mechanisms based on many studies^43,56,99^ as follows:

1. The higher surface area enhances their interactions with surrounding molecules, including enzymes, proteins, and ions.
2. Nano ZrO_2_ has the ability to release Zr^4+^ that can affect several cellular processes, including enzyme activity, cell signalling pathways, and gene expression.
3. Related to their higher reactivity, they can produce reactive oxygen species (ROS) that can lead to microbial cellular damage and lipid peroxidation, which in turn affects the integrity of the microbial membrane.

**Table 6:** Antimicrobial activity of glass and glass ceramic samples (MIC).

Samples code		Inhibition of microbial growth (%)				
		Microbes				
		*Bacillus cereus*	*Staphylococcus aureus*	*E. coli*	*Candida albicans*	*Aspergillus niger*
Glass	BGZr0	34.67 ± 0.09	14.98 ± 0.23	26.45 ± 0.11	26.03 ± 0.16	−
	BGZr1	43.87 ± 0.04	23.15 ± 0.36	27.36 ± 0.24	5.20 ± 0.25	−
	BGZr2	43.30 ± 0.11	28.95 ± 0.17	31.13 ± 0.68	−	−
	BGZr3	51.33 ± 0.39	36.96 ± 0.68	19.06 ± 0.33	−	−
	BGZr4	15.20 ± 0.58	8.36 ± 0.27	11.70 ± 0.47	−	−
Glass ceramic	BGCZr0	49.65 ± 0.25	51.20 ± 0.51	19.56 ± 1.08	26.52 ± 0.25	17.37 ± 0.15
	BGCZr1	49.84 ± 0.59	51.42 ± 0.84	22.40 ± 0.99	39.94 ± 0.85	37.92 ± 0.15
	BGCZr2	50.20 ± 0.75	59.08 ± 0.62	31.23 ± 0.41	53.67 ± 0.34	50.36 ± 0.51
	BGCZr3	51.18 ± 0.67	59.52 ± 0.15	52.37 ± 0.47	45.05 ± 0.59	46.62 ± 0.21
	BGCZr4	56.59 ± 0.14	60.83 ± 0.75	23.34 ± 0.69	38.34 ± 0.57	19.49 ± 0.61

However, all these mechanisms are proposed based on the literature, compositional and structural features of the materials, and require further experimental verification, such as pH and ROS measurements in future work.

#### Longer-term stability assessments

In general, the results obtained in Table 7 exhibited slight decrease in antimicrobial activity of samples stored for approximately 9 months compared to the initial results of freshly prepared samples (Table 6), indicating minor changes over storage time; however, the overall activity recommended stable performance over time and suggested good long-term stability of samples. In this study, no additional characterization (such as XRD, FTIR, or surface analysis) was carried out on the stored samples to confirm the cause of the antimicrobial activity decrease. However, based on the literature, the decrease in antimicrobial activity after long-term storage in different conditions may be caused by surface structure changes, surface relaxation, and the possible loss of surface-adsorbed active ions (e.g., Ca^2^⁺, Zr^4^⁺)^100,101^.

**Table 7:** Antimicrobial activity of glass and glass–ceramic samples after storage on broth media.

Samples code		Inhibition of microbial growth (%)				
		Microbes				
		*Bacillus cereus*	*Staphylococcus aureus*	*E. coli*	*Candida albicans*	*Aspergillus niger*
Glass	BGZr0	25.63 ± 0.29	7.63 ± 0.54	22.96 ± 0.20	20.36 ± 0.12	−
	BGZr1	36.85 ± 0.75	15.97 ± 0.77	23.95 ± 1.05	−	−
	BGZr2	37.92 ± 0.92	21.66 ± 0.51	25.84 ± 0.50	−	−
	BGZr3	45.33 ± 0.77	30.54 ± 0.88	11.23 ± 0.54	−	−
	BGZr4	6.93 ± 0.22	3.00 ± 0.74	6.84 ± 0.85	−	−
Glass ceramic	BGCZr0	45.15 ± 0.86	44.32 ± 0.63	12.00 ± 0.10	14.63 ± 0.48	20.25 ± 0.21
	BGCZr1	44.32 ± 1.06	47.63 ± 0.65	31.65 ± 0.11	17.52 ± 0.11	33.25 ± 0.07
	BGCZr2	46.55 ± 0.38	54.32 ± 1.08	44.54 ± 0.05	25.14 ± 0.25	46.12 ± 0.15
	BGCZr3	46.30 ± 0.78	53.69 ± 0.37	39.51 ± 0.61	46.88 ± 1.15	38.04 ± 0.11
	BGCZr4	50.53 ± 0.25	55.22 ± 0.44	14.05 ± 0.01	16.22 ± 0.63	32.61 ± 0.16

#### Cytotoxicity evaluation

**Fig. 11:**
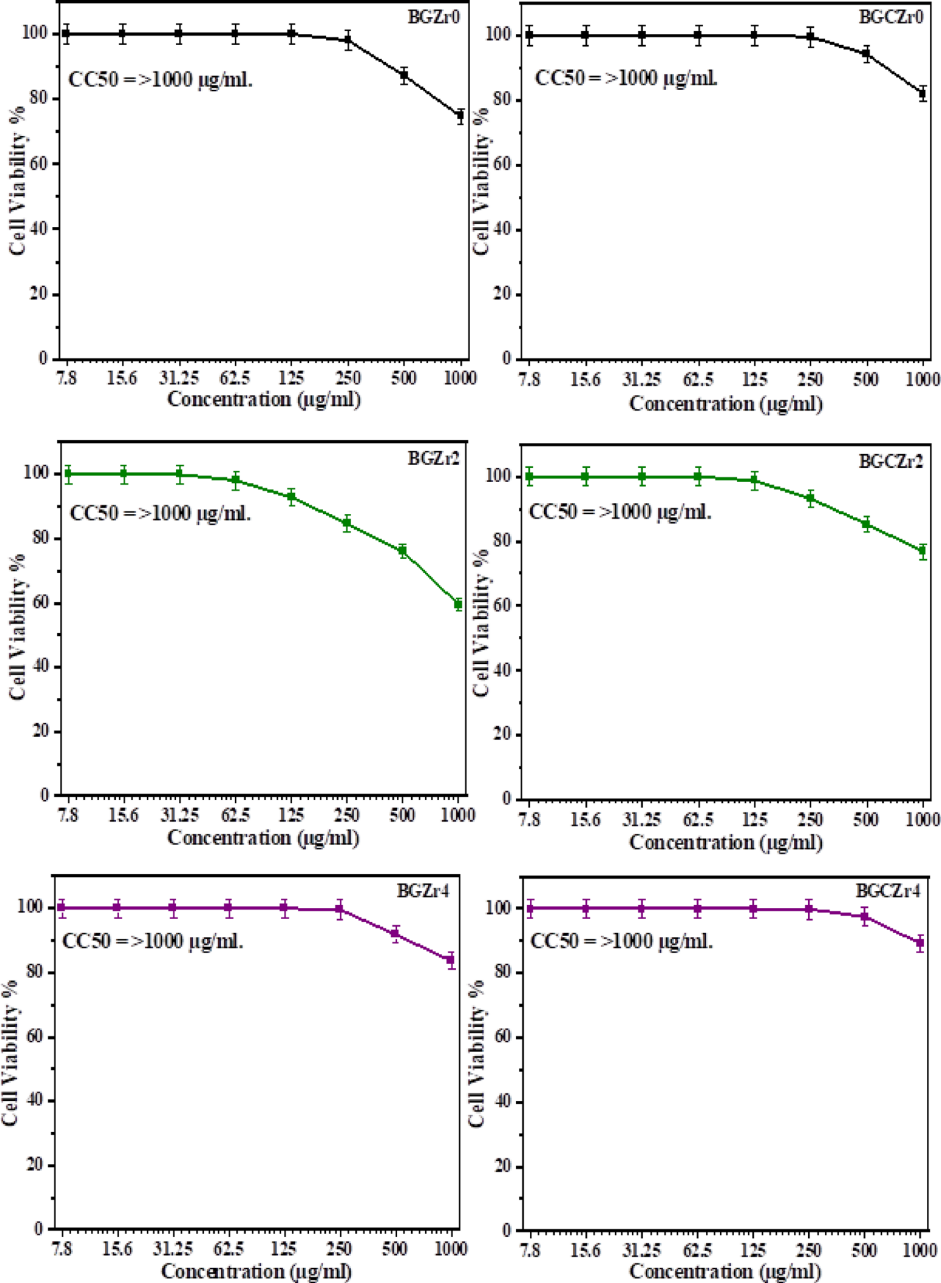
the impact of various concentrations of selected samples of glass (BGZr0, BGZr2, and BGZr4) and glass ceramic (BGCZr0, BGCZr2, and BGCZr4) on Cell Viability % and CC_50_.

Cytotoxicity is an important concern in the development of biomaterials. In order to evaluate the cytotoxicity of the prepared glasses and glass–ceramics, the number of viable cells was determined by the MTT test to assess their biocompatibility. Cell viability % was evaluated using the human normal melanocytes cell line (HFB4) incubated with different concentrations of glass (BGzZr_0_, BGZr_2_, and BGZr_4_) and glass ceramic (BGCZr0, BGCZr2, and BGCZr4) samples for 24 h, as demonstrated in Table 8. Figure 11 displays the HFB4 cell line viability in response to exposure to different concentrations (7.8–1000 µg/ml**)** of the selected samples. Also, the concentration required to cause toxic effects in 50% of intact cells (CC_50_) was included in Fig. 11. It is noticed that all examined samples (except BGZr_2_ and BGCZr2) achieved 100% cell viability at concentrations in the range from 7.8 to 125 µg/ml. From the obtained data, it is clearly observed that:

**Table 8:** Cell viability % values against various concentrations of glass (BGZr0, BGZr2, and BGZr4) and glass ceramic (BGCZr0, BGCZr2, and BGCZr4) samples.

Sample conc. (µg/ml)	Cell viability %					
	Glass			Glass ceramics		
	BGZr0	BGZr2	BGZr4	BGCZr0	BGCZr2	BGCZr4
1000	74.69 ± 1.23	59.54 ± 2.15	83.72 ± 1.23	82.06 ± 1.48	76.81 ± 0.97	89.24 ± 1.75
500	87.15 ± 0.71	76.03 ± 1.79	91.86 ± 0.27	94.17 ± 0.95	85.29 ± 1.43	97.53 ± 0.29
250	98.04 ± 0.28	84.71 ± 1.03	99.62 ± 0.14	99.58 ± 0.24	93.16 ± 0.62	99.87 ± 0.11
125	100	92.85 ± 0.68	100	100	98.74 ± 0.58	100
62.5	100	98.16 ± 0.42	100	100	100	100
31.25	100	100	100	100	100	100
15.6	100	100	100	100	100	100
7.8	100	100	100	100	100	100
0	100	100	100	100	100	100

(I) Glass–ceramics exhibited higher cell viability compared to their parent glasses.

(I) At the same concentration of both glass and glass–ceramic samples, the ZrO_2_-free samples (BGZr_0_ and BGCZr0) displayed higher cell viability compared to 1 mol% ZrO_2_ content (BGZr_2_ and BGCZr2), respectively; however, further increasing ZrO_2_ content to 2 mol% (BGZr_4_ and BGCZr4) caused cell viability improvement.

Since the cytotoxicity is related to the ion-exchange reactions upon contact with the cell culture medium^97^, the observed cell viability improvement by increasing ZrO_2_-content can be attributed to the reduction of released ions due to the decreased solubility upon the simulation of crystallization by increasing ZrO_2_-content in the studied samples. These obtained results confirm the non-toxic nature and the biocompatibility of the tested samples, even at higher concentrations because the cell viability is greater than 70%^102^.

Overall, the dielectric, antimicrobial and biological response of our samples is consistent with earlier findings for ZrO_2_-doped borate glasses^76–80,97,98^, where Zr^4+^ ions improve biocompatibility, stability, and antimicrobial performance. Accordingly, the enhanced dielectric and biological performance make our samples comparable or superior to existing glass systems, confirming their potential in biomedical and electronic applications.

## Conclusion

In this study, ZrO_2_-doped borate glass of composition (40 B_2_O_3_–20 Al_2_O_3_–10 LiF-(30-X) CaO–X ZrO_2_) mol% where (0 ≤ X ≤ 2.5) mol%, was synthesized by the melt quench method to evaluate the influence of the gradual increase of ZrO_2_ content in the glass matrix on structure, electrical properties, and antimicrobial activity. Additionally, the corresponding glass–ceramics were prepared by heat treatment at nucleating temperature (500 °C) with a rate of 5 °C/min for 4 h. Then, the temperature was elevated to the crystal growth temperature (725 °C) with a rate of 5 °C/min for 10 h. XRD analysis confirmed the formation of the nanocrystalline ZrO_2_ phase within the glass–ceramic matrix. The average crystallite size, determined by the Scherrer formula, fell within the range (23–79) nm. According to the FTIR study, ZrO_2_ acts as a network modifier in this system, where zirconium ions in the glass structure induce the BO_4_ → BO_3_ back conversion, resulting in an increase of non-bridging oxygen (NBO_S_). The density of the glass rises from 2.5892 to 2.6159 g/cm^3^ by the substitution of CaO by ZrO_2_, while the molar volume increases by increasing ZrO_2_ content in this glass system from 26.1305 to 26.3775 cm^3^/mol due to the formation of NBO_S_. Dielectric and conductivity studies reveal that ZrO_2_ addition enhances permittivity (ε′) and AC conductivity (σ) while reducing dielectric losses due to creating NBOs in both glass and glass–ceramic samples that enhance polarizability and promote ion transport. Glass–ceramics show enhanced dielectric properties compared to glasses due to the formation of crystalline phases (α-CaAl_2_B_2_O_7_, Nano-sized ZrO_2_, and CaB_2_O_4_) and reduced losses. Frequency-dependent behaviour confirms hopping conduction and space charge effects, with electric modulus analysis effectively distinguishing overlapping relaxation processes. Both glass and glass–ceramics samples exhibit antimicrobial responses depending on the concentration of ZrO_2_ content in the glass matrix, but the antimicrobial activity of glass–ceramics is more than that of their parent glasses due to the separation of the nanocrystalline ZrO_2_ phase, which acts in a similar manner to ZrO_2_ nanoparticles. Cytotoxicity results confirm the non-toxic nature of the prepared samples even at higher concentrations. long-term stability results exhibited slight decrease in antimicrobial activity of samples stored for approximately 9 months compared to freshly prepared samples. Finally, the study investigated the suitability of the prepared materials in both electrical and biomedical applications.

## Data Availability

The data of this work is available for any person after publication. For any questions about the data from this study contact the corresponding author (Gehad Y. Abo El-Reesh).
